# Acute Scrotal Abdomen: An Epitome of Negligence in Rural India

**DOI:** 10.7759/cureus.24784

**Published:** 2022-05-06

**Authors:** Dharmendra K Pipal, Abdul Waseem Khan, Vijay Verma, Seema Yadav, Lakhan Kumar Purohit

**Affiliations:** 1 General, Colorectal and Minimal Access Surgery, All India Institute of Medical Sciences, Gorakhpur, Gorakhpur, IND; 2 General Surgery, Dr. Sampurnanand Medical College, Jodhpur, IND; 3 Anaesthesia, Jaipur National University Medical College, Jaipur, IND

**Keywords:** giant inguinoscrotal hernia, mortality, septic shock, peritonitis, transverse colon perforation

## Abstract

An inguinoscrotal hernia is considered to be giant when it passes beyond the midpoint of the thigh in a standing position. It is a rare condition that can lead to complications such as obstruction and perforation. Here, we present the case of a 35-year-old male who was diagnosed with a giant inguinoscrotal hernia with transverse colon perforation peritonitis. The patient presented with acute abdomen and septic shock. On presentation, resuscitation was started and an emergency laparotomy was performed. Resection of the gangrenous bowel segment and end jejunostomy was done as damage control surgery. However, despite intensive care and efforts, the patient succumbed due to multiorgan dysfunction syndrome (MODS). This is a rare case of a giant inguinoscrotal hernia with transverse colon perforation peritonitis, leading to MODS and mortality.

## Introduction

A giant inguinoscrotal hernia [1] is a hernia that extends beyond the mid-point of the thigh observed while the patient is standing. Although gut loops are the most frequent contents of such large hernias, a literature search reveals several case reports mentioning the stomach, duodenum, ovaries, and even the ureter and bladder as hernial contents. Although the possibility of incarceration of hernia is very low, ranging from 0.3% to 3% per year, its long-term complications often culminate in even more life-threatening complications, such as strangulation and perforation of its content if it is the gut [1]. In such circumstances, intervention can result in a high rate of morbidity and mortality [2]. In this report, a case of a gigantic, strangled inguinoscrotal hernia with perforation peritonitis is discussed.

## Case presentation

**Figure 1:**
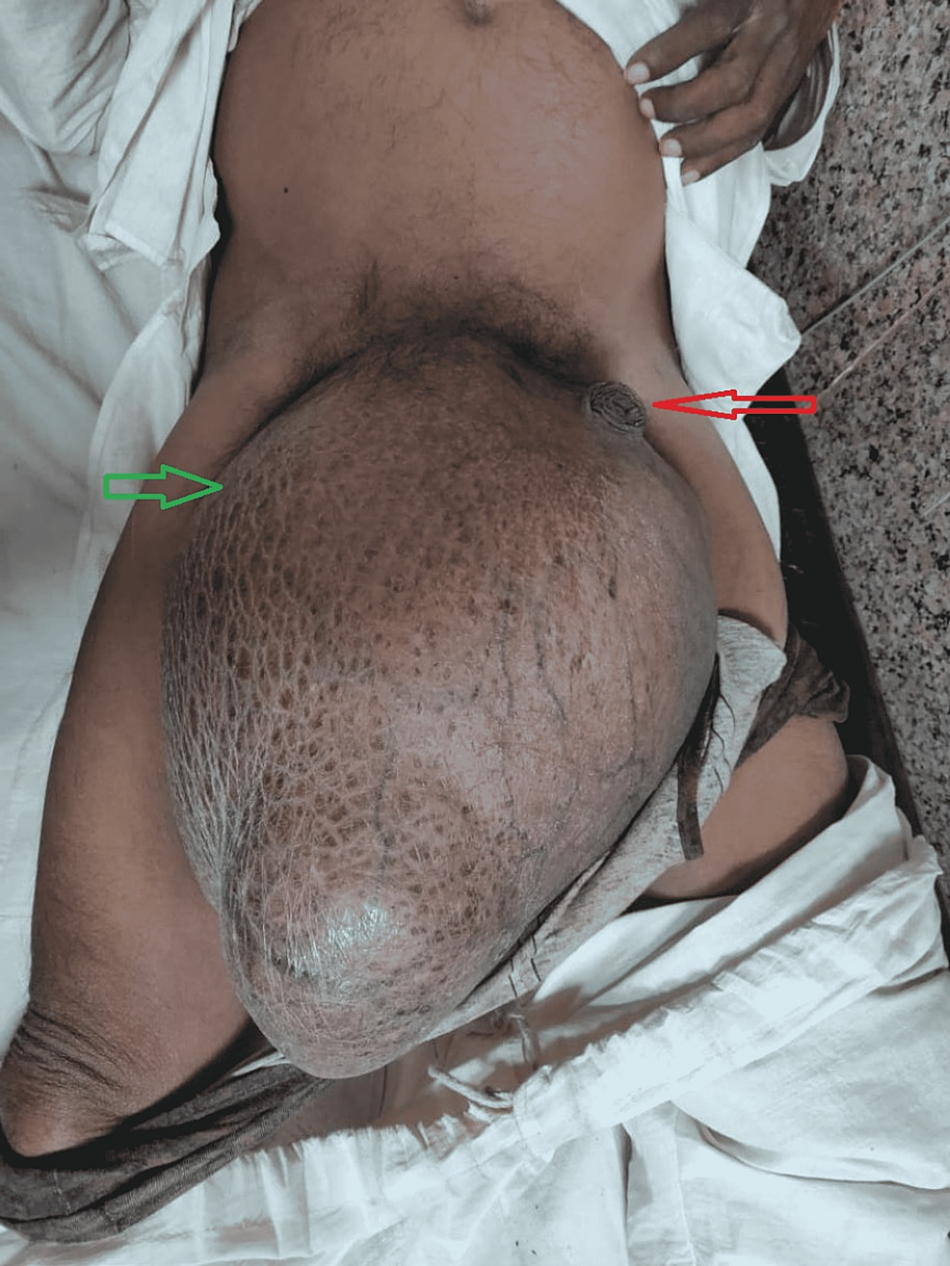
Giant inguinal hernia. The green arrow denotes the huge hernial swelling reaching up to the knee, and the red arrow denotes the buried penis.

A 35-year-old male, a chronic alcoholic, smoker, and drummer by occupation, from rural India, presented to the surgical emergency of Dr. Sampurnanand Medical College, Jodhpur, Rajasthan, with a 10-year history of an enormous swelling in the groin and scrotum and abdominal pain for the last 10 days. He had visited casualty 15 days before but was declined admission and surgery. The pain was constant and burning, radiating all the way down from the abdomen to the scrotum. Unreducible, tender, and without any cough impulse or transillumination, a huge inguinoscrotal swelling extending nearly to the knee was noted on examination (Figure 1). The penis was buried, and the penile skin was stretched. The abdomen was distended, with generalized discomfort, guarding, and rigidity.

The patient had tachycardia with a pulse rate of 130 beats per minute at the time of admission. The blood pressure was 80/60 mmHg. The patient was tachypneic (respiratory rate of 32breaths per minute) and SpO_2_ of 96% with a venturi mask. An X-ray revealed a gas bubble underneath the diaphragm. Using intravenous (IV) fluids and inotropes, the patient was resuscitated. Antibiotics were administered intravenously. An ultrasound of the abdomen and scrotum demonstrated free fluid in the abdomen and scrotum along with dilated bowel loops.

**Figure 2:**
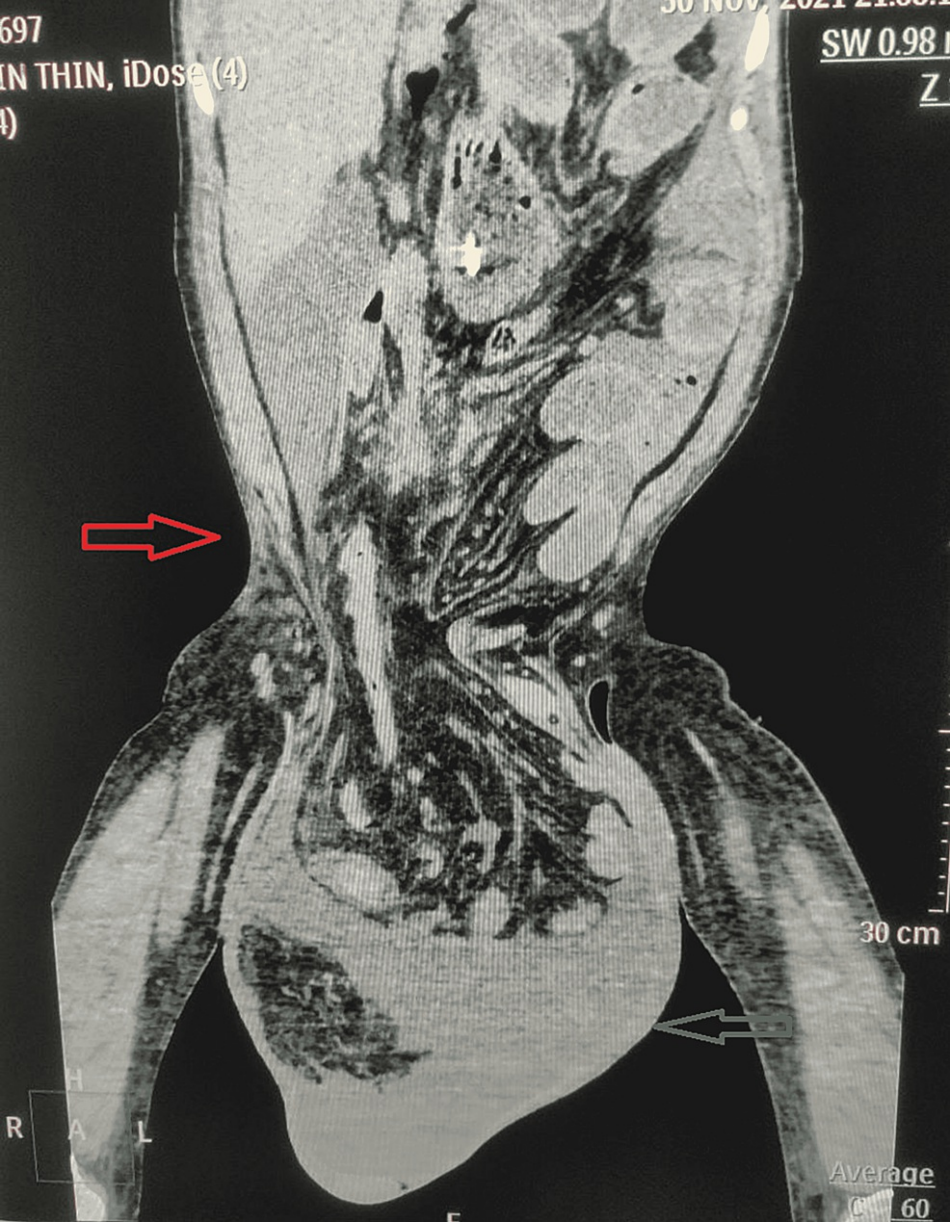
Noncontrast CT coronal view depicting herniation of the abdominal contents into the scrotum. CT: computed tomography

**Figure 3:**
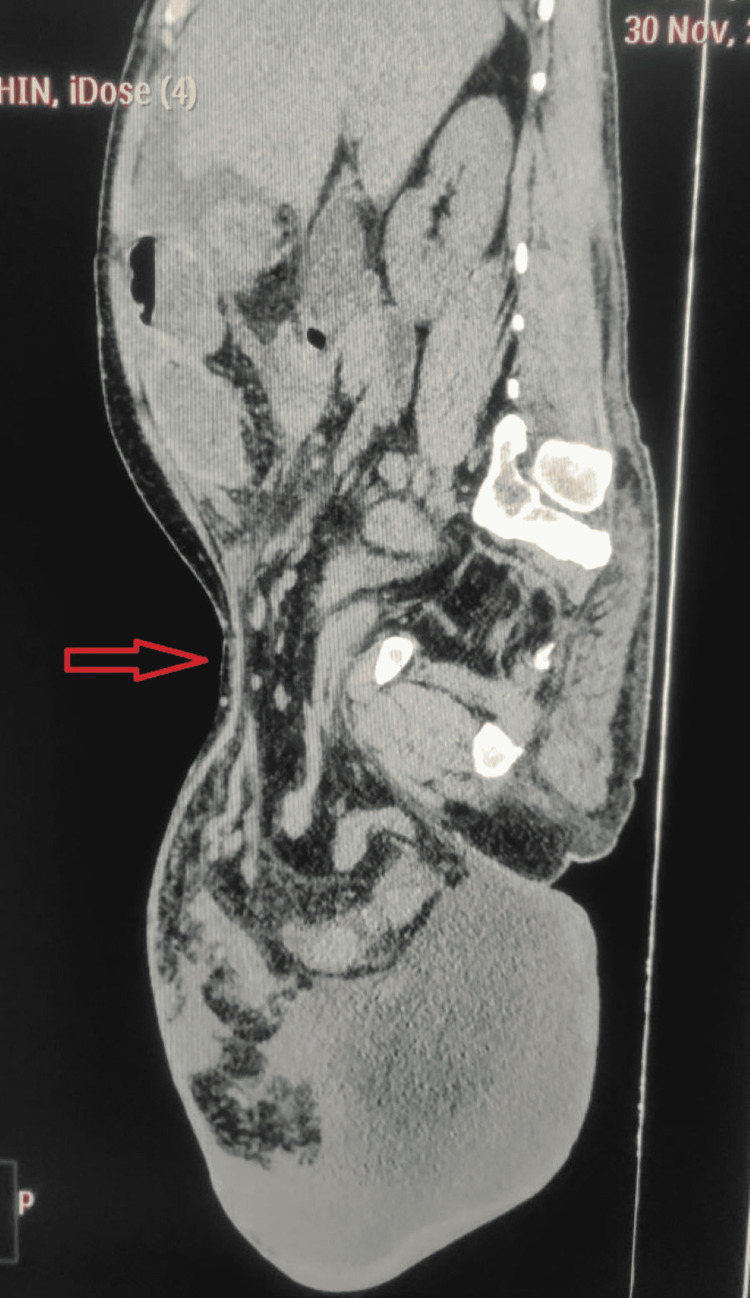
Noncontrast CT sagittal view depicting herniation of the abdominal contents into the scrotum. CT: computed tomography

Noncontrast computed tomography (CT) (Figures 2, 3) demonstrated free fluid with pneumoperitoneum both in the abdominal cavity and the inguinoscrotal area. Additionally, the scrotum contained bowel loops extending from the mid-jejunum to the ascending colon.

Under general anesthesia, the patient was operated through an oblique scrotal as well as a midline laparotomy incision. The entire bowel, from the jejunum to the right transverse colon, including the omentum, was located inside the scrotal sac. A total of 15 L of fecal-infested fluid was evacuated. Due to compression at the neck of the hernial sac, the small bowel mesentery and mesocolon became thrombosed. The proximal part of the transverse colon and a substantial amount of the small intestine extending from the mid-jejunum to the ileocaecal junction was found to be gangrenous. Caecum had patchy pre-gangrenous alterations as well. An end jejunostomy (Figure 3) was performed after resection of the complete intestine from the mid-jejunum to the mid transverse colon.

**Figure 4:**
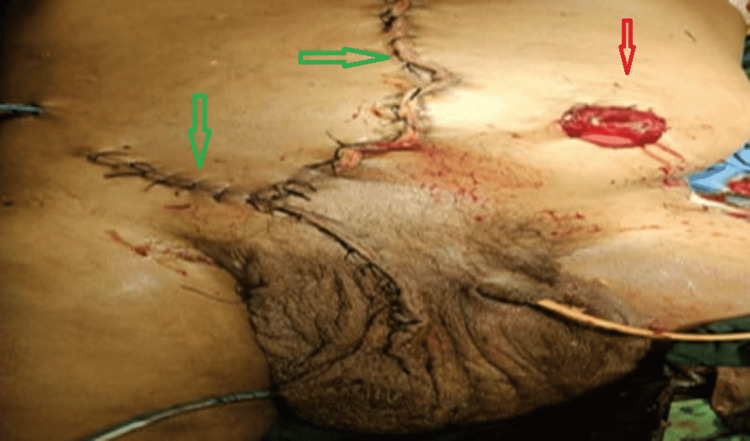
Immediate postoperative image. Red arrow- Depicting jejunostomy Green arrow- Depicting laparotomy and inguinoscrotal wound

An inguinal herniorrhaphy was performed, and the scrotum was left alone with a drain in situ. The patient was kept intubated and monitored in the intensive care unit following the surgery. On the first postoperative day, the patient was kept on ventilatory support (pressure control mode) with a heart rate of 130 beats per minute and blood pressure of 90/70 mmHg on noradrenaline support at 2 amps and 10 mL/hour.

**Table 1:** Laboratory parameters.

Parameter	At the time of admission	Postoperative day one	Reference value
Hemoglobin	11.6	9.2	11.6–15.0 g/dL
Total leucocyte count	6,103	22,103	4,000–11,000/µL
Blood urea	70	102	17–43 mg/dL
Serum creatinine	1.48	5	0.6–1.2 mg/dL
Serum glutamic-oxaloacetic transaminase	57	450	10–40 U/L
Serum glutamic pyruvic transaminase	20	560	8–56 U/L
Serum albumin	2.8	2.0	3.5–5.5 g/dL

The patient developed multiorgan dysfunction syndrome (MODS), as depicted in Table 1. Despite all intensive efforts and care, the patient’s condition deteriorated and he succumbed on postoperative day two.

## Discussion

Overall, 75% of abdominal wall hernias occur in the inguinal region, with a lifetime incidence of 27% in men and 3% in women. It is classified as a direct or indirect hernia based on the location of the bulge in the inguinal canal. It is the most frequent type of hernia in men and is associated with a higher risk of problems as time goes on [3,4]. Illiteracy, fear of mortality, and carelessness combined result in a severe hazard. Additionally, due to technical challenges, such as cardiorespiratory insufficiency, sometimes surgeons may advocate against surgical intervention [5]. Patients with a large inguinoscrotal hernia experience difficulties such as walking, sitting, and performing daily activities. Nevertheless, they do not seek surgical treatment. Additionally, patients with these hernias experience severe morbidities, including voiding difficulties, urine retention, scrotal skin thickening, and testicular atrophy. The mesentery, small bowel, appendix, caecum, colon, kidney, ureter, and bladder are all possible contents of a hernia sac. Strangulation, obstruction, and perforation can occur in irreducible hernias [6-10]. Moreover, constant downward pressure can cause perforation [6]. A loss of domain is also common in this form of hernia, necessitating a multidisciplinary approach during treatment.

Because of the protracted nature of these massive inguinoscrotal hernias, the abdominal cavity becomes empty and loses capacity, causing a dramatic rise in pressure in the abdominal and intrathoracic cavities on sudden replacement of the intestine from the scrotum to the abdomen, as seen commonly after surgery [10]. Increased pressure can hinder diaphragmatic movement and diminish venous return, leading to compartment syndrome. Furthermore, abdominal distention causes strain on the surgical wound, which slows delayed healing.

In several case studies, different procedures, such as hemicolectomy, omentectomy, resection of the small bowel, and splenectomy, have been mentioned as various treatment options for enormous inguinoscrotal hernias [11]. The postoperative recovery in these patients is marked by a prolonged period of elective mechanical breathing. Therefore, ventilation in the ICU settings for a minimum of 10 days is recommended.

The poor general condition, toxemia, and gangrenous gut necessitated small bowel resection and partial colectomy in our patient, followed by an end jejunostomy. Given our patient’s perforation and bowel contents spilling during surgery, the possibility of domain loss was doubtful. Despite early surgery and rigorous sepsis treatment, we were unable to save the patient. The patient most certainly had a huge inguinal hernia for many years. His initial labs and vitals revealed that he possibly presented shortly after perforation. This case demonstrates that despite early and intensive treatment, a strangulated hernia perforation into the scrotum has a very high fatality rate.

Resection of the mesentery, small bowel, total or hemicolectomy, and reconstruction techniques can all be performed during surgery. To correct domain loss, laparostomy with Bogota bag placement, component separation technique, and other techniques can be used [12]. For cosmetic reasons, excess scrotal skin might be removed, and a scrotum reconstruction can be performed [13]. However, leaving the scrotum intact is advantageous because if intra-abdominal pressure rises, the content can be decreased back into the scrotum. To prevent seroma and hematoma, a drain should be put into the scrotum at the end of the surgery, which can be removed later [14].

## Conclusions

An uncomplicated giant hernia should be managed via proper preoperative workup after a thorough cardiopulmonary and coagulation profile assessment; however, in emergencies, such as strangulation with peritonitis and shock, mortality is very high, as seen in our case. Therefore, negligence at any level must be eliminated, be it at the patient’s or the primary healthcare physician’s level. Counseling patients regarding getting operated on at the earliest plays a pivotal role in preventing mortality in these patients.
